# Herpes-Zoster-Associated Voiding Dysfunction in an Immunocompromised Patient

**DOI:** 10.7759/cureus.8469

**Published:** 2020-06-06

**Authors:** Adel A Alalwan, Alaa Ali

**Affiliations:** 1 Nephrology, Salmaniya Medical Complex, Manama, BHR; 2 Pediatric Surgery, Salmaniya Medical Complex, Manama, BHR

**Keywords:** herpes zoster, voiding dysfunction, urinary retention, immunocompromised patient

## Abstract

During herpes zoster infection, immunocompromised hosts are especially vulnerable to complications, which include visceral organ involvement. Voiding dysfunction secondary to herpes zoster infection is an uncommon clinical presentation and has numerous enigmatic mechanisms. This case of herpes-zoster-associated voiding dysfunction occurred in a patient with nephrotic syndrome treated with immunosuppressives (prednisolone and mycophenolate mofetil). The patient presented with acute urinary retention and extensive lumbosacral herpetic infection. He responded positively to treatment and completely recovered following a 14-day course of intravenous acyclovir and intermittent self-catheterization (two to three times daily) for four weeks.

## Introduction

Herpes zoster is a common disease caused by the varicella-zoster virus (VZV), the culprit in two clinically distinct diseases. After the initial infection with VZV, varicella (chickenpox) occurs and is characterized by widespread vesicular lesions in various stages of development. Herpes zoster, also called shingles, results from the reactivation of latent VZV within the sensory ganglia. The classical presentation of the disease is characterized by a painful, unilateral vesicular eruption occurring in a restricted dermatomal distribution [1,2]. Immunocompromised hosts, in particular, are at considerable risk for atypical presentations of the disease as well as other systemic manifestations and visceral organ involvement such as bladder and anal sphincter dysfunction [3,4]. Voiding dysfunction is a rare clinical presentation, and its description in the literature is scarce, leaving its mechanisms largely misunderstood.

We report a case of herpes-zoster-associated voiding dysfunction in a patient with nephrotic syndrome, treated using immunosuppressive therapy, and describe its mechanisms, clinical presentation, and course of treatment.

## Case presentation

A 36-year-old man with nephrotic syndrome treated with immunosuppressive therapy presented with acute urinary retention and a three-day history of lower abdominal discomfort. He was unable to void completely despite straining. These complaints were preceded by the eruption of a vesicular erythematous rash over the right side of his lower abdomen which extended to his right scrotum and thigh. The rash appeared approximately seven days before the voiding complaints and was not associated with pain or itching. He did not have similar symptoms previously. He had no fever and no other urinary symptoms such as dysuria, urgency, and increased frequency. He denied a history of lower back pain, abnormal sensation in the legs, and weakness and did not have constipation or loss of sexual ability. He had a history of childhood chickenpox.

The patient had a known history of nephrotic syndrome with stage III chronic kidney disease for two years. Although his renal biopsy was suggestive of minimal change disease with mesangial immune complex deposits, another biopsy performed five months prior confirmed a diagnosis of the tip lesion variant of focal segmental glomerulosclerosis. His medications were prednisolone (15 mg daily) and mycophenolate mofetil (1000 mg twice daily), both for approximately six months. These were not altered at admission.

**Figure 1:**
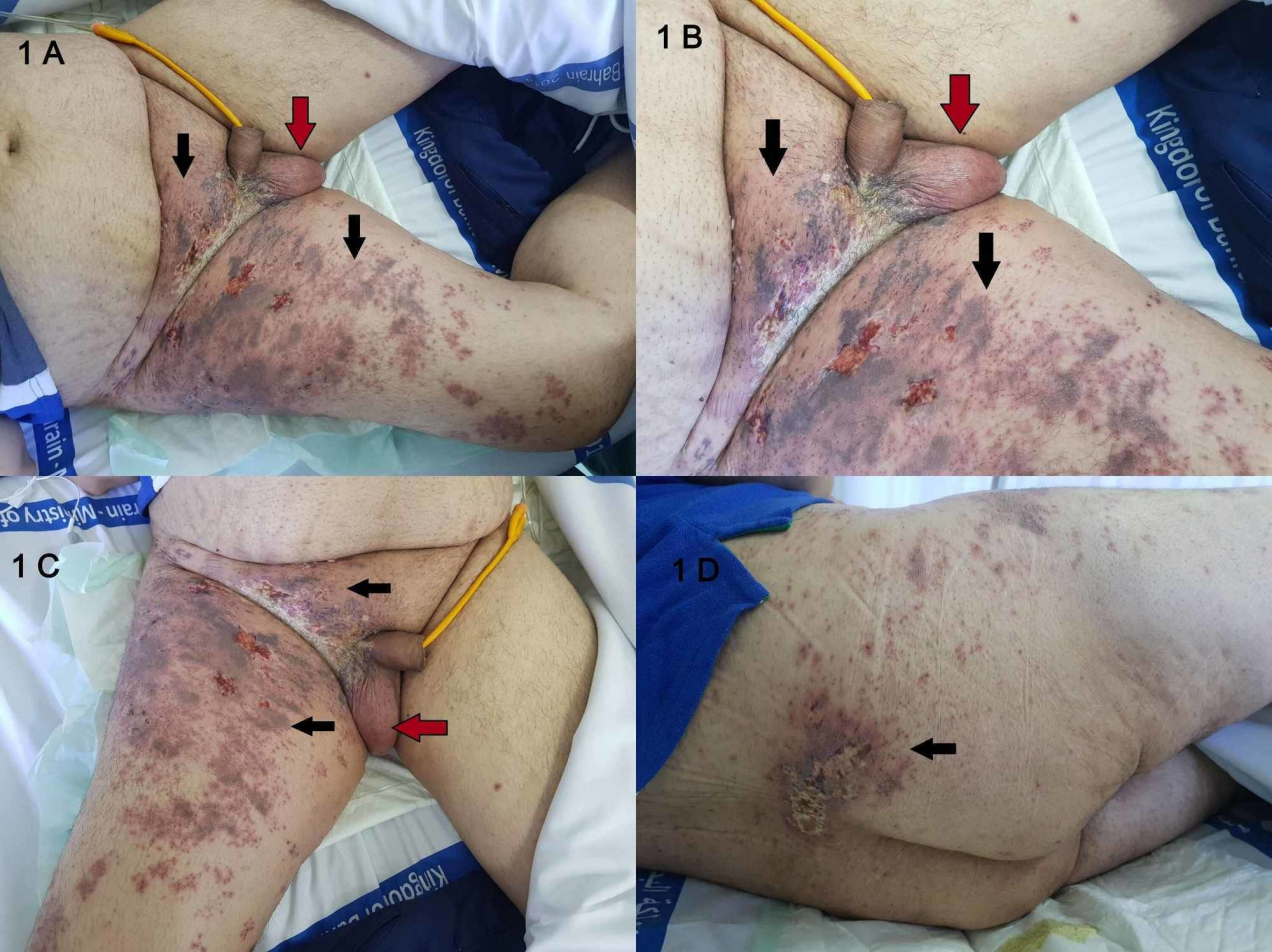
Figures A, B, C - Front View. Figure D - Back View On admission: Vesicular rash on a densely erythematous base with areas of purpura involving the L1 to L4 dermatomes (black arrows). The ruptured vesicles have led to the development of ulcerations with crusted lesions in the same distribution. S2 dermatome is involved as evidenced by the right scrotal rash and erythema (red arrows).

The patient was vitally and clinically stable at admission. He had central obesity with corticosteroid-related cushingoid features such as a moon-like face, suprascapular fat pads, and abdominal striae. He had suprapubic tenderness, and his bladder was palpable above the symphysis pubis. There was a non-tender vesicular rash on a densely erythematous base with areas of purpura involving the L1 to L4 dermatomes (Figures 1A-1D). The ruptured vesicles led to the development of ulcerations with crusted lesions of the same distribution. The rash also involved the S2 dermatome as evidenced by the right scrotal rash and erythema (Figures 1A-1C). The patient’s respiratory and cardiovascular examinations were unremarkable, and a neurological examination showed no weaknesses in the lower limbs; sensation was intact. Deep tendon reflexes and anal sphincter tone were preserved.

Laboratory tests showed that his white blood cell count was 6.08 x 10^9^ per L, hemoglobin was 9.4 g/dL, platelet count was 198 x 10^9^ per L, and random blood glucose was 6.5 mmol/L. His urea (19.1 mmol/L), creatinine (188 µmol /L), and electrolytes were within his baseline. Creatinine clearance was 40.88 mL/min, and his serum albumin was 24 g/L. Urinalysis showed 3+ proteinuria, no hematuria or pyuria, and no nitrates or glucose. Urine bacterial culture was negative as was urine cytology. His chest X-ray was normal with no interval changes.

An indwelling catheter was inserted to relieve the acute urinary retention, yielding a residual vesical volume of 800 mL. Postcatheterization, a kidney, ureter, and bladder ultrasound was performed, revealing a normal-sized prostate gland (20 mL) and no hydroureter or hydronephrosis. Cystoscopic findings were normal with no signs of urethral obstruction, strictures, or inflammatory changes. The bladder appeared normal with no evidence of cystitis. Urodynamic studies were not available at our facility.

**Figure 2:**
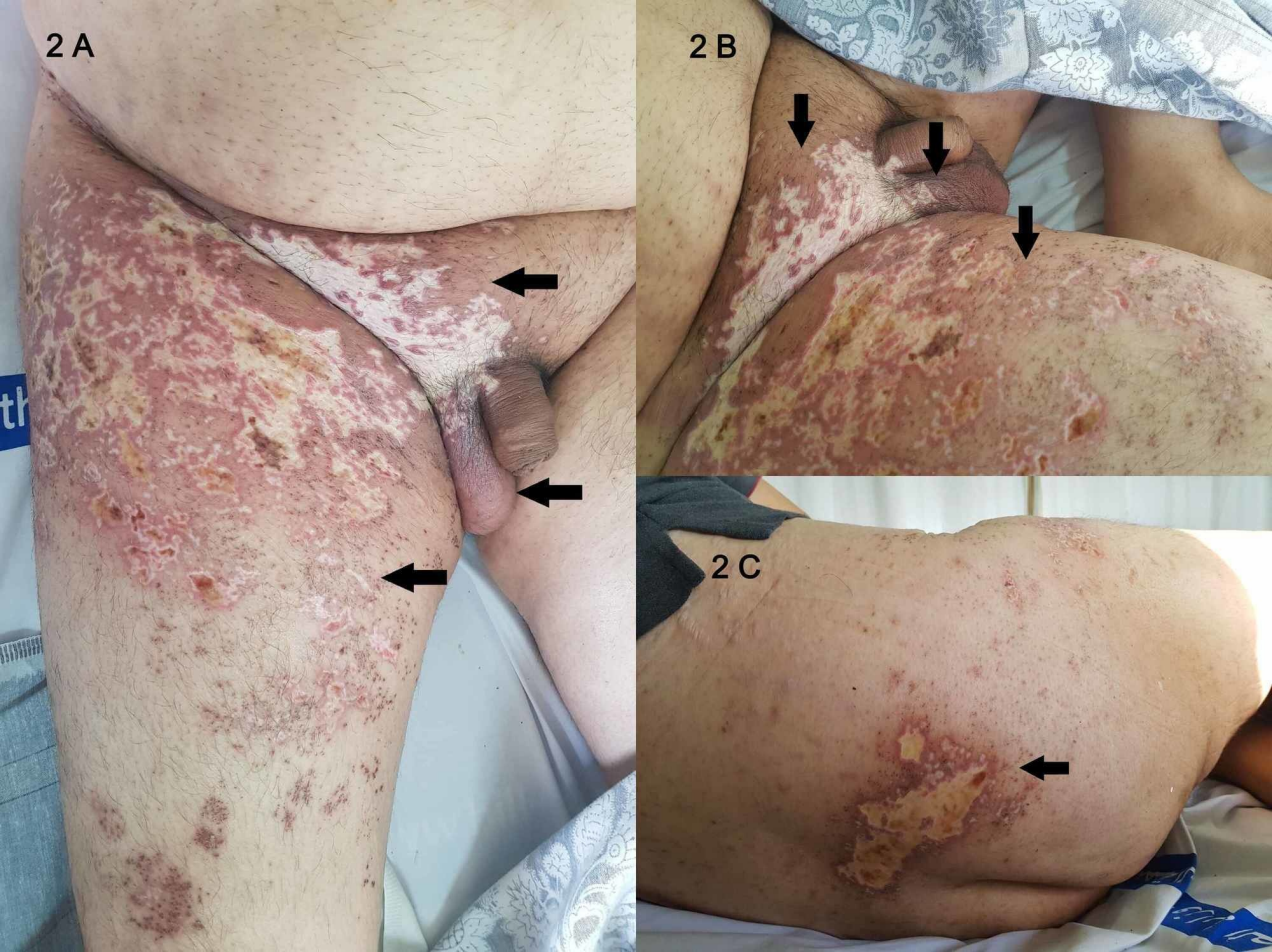
Figures A, B - Front View. Figure C - Back View Day 10 of IV acyclovir: The herpetic rash has significantly improved and in various stages of healing with areas of scarring and hypopigmentation, the lesions are well-demarcated with erythematous hyperpigmented borders (black arrows).

**Figure 3:**
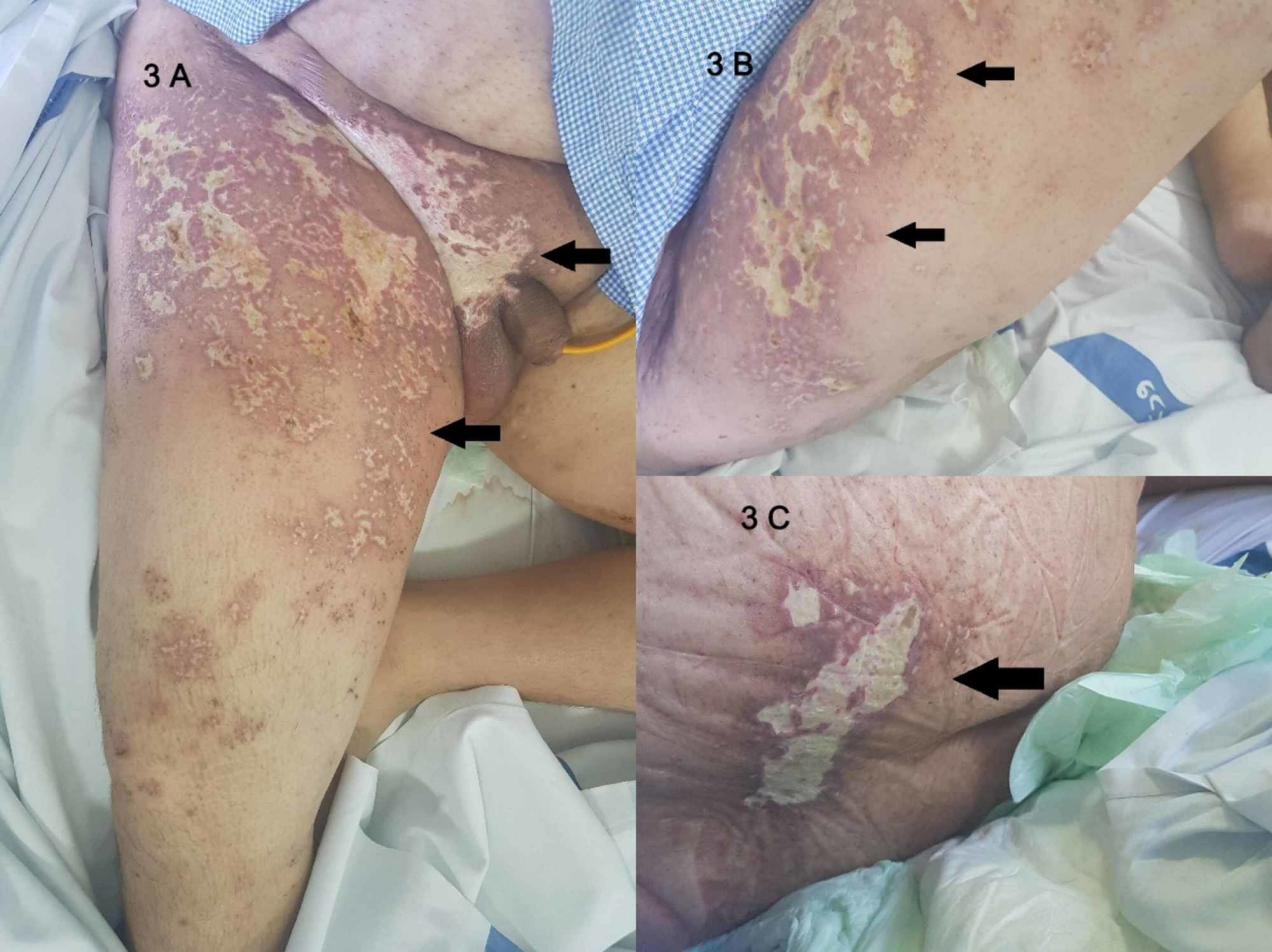
Figure A - Front View. Figure B - Right Side View. Figure C - Back View Day 14 of IV acyclovir: The herpetic rash has healed with areas of scarring and hypopigmentation, the hyperpigmented borders are fading out (black arrows).

The diagnosis was voiding dysfunction secondary to extensive lumbosacral herpes zoster infection. A 14-day renal dose of intravenous acyclovir was started. The improvement was remarkable after 10 days, reducing the rash to well-demarcated areas of scarring and hypopigmentation (Figures 2A-2C) (Figures 3A-3C).

During the hospital stay, adequate urine output was maintained through an indwelling urinary catheter. A trial without a catheter was unsuccessful after the patient developed abdominal discomfort and could not empty his bladder completely despite straining and having an urge to void.

The patient was discharged with instructions for intermittent self-catheterization (two to three times daily) and close follow-up at the nephrology and urology outpatient clinics. Four weeks later, the voiding dysfunction resolved completely, and the patient was able to void spontaneously without the need for self-catheterization. On follow-up, he did not report abdominal discomfort, urgency, increased frequency, or post-void dribbling.

## Discussion

Voiding dysfunction is a broad term used to describe poor coordination between the bladder muscle and urethra. Its causes vary and are classified as obstructive, infectious, inflammatory, iatrogenic, or neurologic, among others. Common causes include benign prostatic hyperplasia, prostatitis, cystitis, receiving anticholinergic or alpha-adrenergic agonist medications, and cortical, spinal, or peripheral nerve lesions. A comprehensive history, physical examination, and selected diagnostic testing should identify the cause of voiding dysfunction in most cases [5].

Herpes zoster is an uncommon cause of voiding dysfunction. It was first reported by Davidsohn in 1890 [6,7]. In 2002, Chen et al. reviewed 423 cases of herpes zoster in a large university-affiliated medical center in Taiwan, finding 17 (4.02%) with voiding dysfunction attributed to herpes zoster. Urination difficulty found in 12 (70.6%) of those 17 patients was clinically classified as cystitis-associated voiding dysfunction. Notably, more than 25% of those with herpes zoster involving lumbosacral dermatomes developed voiding dysfunction [8].

Three primary mechanisms have been described in the etiology of voiding dysfunction associated with sacral herpes zoster. They differ in clinical presentation and course progression. One is ipsilateral hemi-cystitis in which the virus directly invades and replicates in the bladder wall, usually concurrent with the skin rash, sometimes producing dysuria, hematuria, increased frequency, and rarely urinary retention. Another is neuritis-associated voiding dysfunction (flaccid bladder) in which bladder atonia is triggered by an interruption in the detrusor reflex. Detrusor areflexia, triggered by VZV spreading to the peripheral nerves, the sacral motor neurons, or their roots, can be confirmed through urodynamic studies. It typically presents as acute urinary retention four to nineteen days after the skin rash, and the clinical course can last four to eight weeks. Finally, myelitis-associated voiding dysfunction (spastic bladder) is less prevalent; the majority of patients achieve complete recovery after a few weeks [6,8,9].

Non-sacral herpes zoster can also be associated with voiding dysfunction, especially when it directly involves the upper lumbar vertebra (L1-L2). Dales and Wilson suggested a mechanism by which herpetic infection of the upper lumbar segments spreads to the adjacent spinal cord and involves the sympathetic motor fibers to the bladder trigone. Urine retention associated with L1-L2 infection was a possible result of trigone paresis [10]. Our patient’s neuritis-associated voiding dysfunction (flaccid bladder) was most probably the result of S2 dermatomal involvement; trigone paresis is another possible cause, given the L1-L2 dermatomal involvement.

Recovery from herpes-zoster-associated voiding dysfunction can usually be achieved by treating the viral infection, performing intermittent self-catheterization, and controlling pain if required. Complete recovery spans up to 10 weeks [8,11].

Antiviral therapy must be initiated in all immunocompromised patients with herpes zoster, even if presentation is more than 72 hours after onset. Intravenous acyclovir therapy is an effective therapeutic approach in these cases [12]. Immunocompromised patients are particularly susceptible to increased risk of herpes zoster as a result of reduced T-cell-mediated immunity, which predisposes patients to repeated episodes of herpes zoster infection with unconventional clinical presentations and VZV-related complications such as cutaneous dissemination and visceral organ involvement [4,13].

## Conclusions

Awareness and vigilance are vital when addressing herpes zoster because visceral organ involvement such as voiding dysfunction must be promptly recognized for proper diagnosis and management, especially in immunocompromised patients. Voiding dysfunction is a rare but important complication of lumbosacral herpes zoster, and it has a variety of mechanisms. In cases of urinary retention, an effective treatment plan includes antiviral therapy, urethral catheterization in the acute setting, and a course of intermittent self-catheterization in the out-patient setting. Because herpes-zoster-associated voiding dysfunction runs a relatively short and benign course in most cases, major urological symptoms will resolve if other causes are ruled out.
